# Understanding Facilitators and Barriers of Online Cancer Information Utilization among Cancer Survivors and their Families: Focus on the Theory of Planned Behavior

**DOI:** 10.31557/APJCP.2020.21.5.1357

**Published:** 2020-05

**Authors:** Dong-Soo Shin, Saerom Saerom, Heui-Sug Jo

**Affiliations:** 1 *Division of Nursing, Research Institute of Nursing Science, Hallym University, Republic of Korea, *; 2 *Department of Health Management and Policy, School of Medicine, Kangwon National University, Republic of Korea. *

**Keywords:** Cancer survivors, the internet, health information, theory of planned behavior

## Abstract

**Objective:**

Cancer survivors have various health care needs and are willing to be proactive with their health maintenance. Online information would be a useful resource to guide cancer survivors and their family members. Therefore, identifying the factors that influence Internet searching behaviors among cancer survivors and their family members is a first step toward providing better health care services for cancer care.

**Methods:**

We performed focus group interviews that were based on the Theory of Planned Behavior, with thirty-one participants to explore factors related to Internet search behaviors among cancer survivors and their family members.

**Results:**

Six themes were identified in the analysis of participant interviews. Attitudes toward searching for health information on the Internet included the themes “*Fulfilling unmet needs*” and “*Confirmation through second opinion*.” Themes related to social norms included “*a required step for sure*” and “*helping each other*.” In terms of perceived behavioral control, themes included “*difficult to choose because of being* ‘*overwhelmed with information*,’” and “*complex searching milieu*.”

**Conclusion:**

It was clear that cancer survivors and their family members had unmet needs for maintaining their health status. They wanted to be informed and actively involved in the decision-making process regarding health management. Consultation and education provided to patients by doctors should not only include information on diet and nutrition but also information on the resulting complications to satisfy their need for reliable health information**.**

## Introduction

Cancer survivors may experience various physical and psychosocial health care needs. These may include late complications, chronic illnesses, depression, and anxiety from a possible occurrence of a second cancer diagnosis (Shin et al., 2015). The Chronic Care Model (CCM) emphasizes the importance of the interaction between an informed active patient and a prepared, proactive practice team, to improve the functional and clinical outcomes of patients with chronic disease. Therefore, the doctor’s and patient’s health literacy is an essential factor in improving patient outcomes, in addition to enhancing community resources, health system support, self-management support, delivery system design, decision support, and clinical information systems (Wagner, 1998). 

Cancer survivors and their family members tend to use the Internet to search for health information and connect with other cancer survivors (Yli-Uotila et al., 2013). Utilizing the Internet to obtain health information could further facilitate communication with health care professionals (Tan and Goonawardene, 2017). However, there are concerns related to the quality of health information on the Internet due to variations in quality between resources (Fahy et al., 2014). When individuals with cancer and their family members visit websites to search for health information, there is a high possibility that they retrieve unregulated or unwanted information (Cho and Woo, 2007). In one study, 39.1% of respondents with cancer recognized that the health information from the Internet was not correct (Lee et al., 2014a). 

Consequently, some people avoid using the Internet to search for health information. The result of a preliminary study investigating factors related to health information retrieval among 123 Korean cancer survivors showed that 23% were information-averse and did not use this information. In addition to being information-averse, reasons for avoiding information included information inaccuracies (39.1%), unnecessary information (17.4%), and lack of customized information (17.4%) (Lee et al., 2014b). The above findings highlight the significance of well-developed health information resources that meets the needs of cancer survivors and their families, ensuring the right health information is made available to them. Therefore, identifying the factors that influence Internet searching behaviors among cancer survivors and their family members is a first step towards providing better health care services for cancer care. 

In order to understand the Internet searching behavior of cancer survivors and their family members, we applied the theory of planned behavior (TPB), which provides a framework to understand and explain behavior based on a person’s attitudes, social norms, and perceived control (Ajzen, 1991). Based on this theory, we aimed to explore why cancer survivors and their family members visit the Internet to search for health information and how they perceived their searching behaviors. Furthermore, we examined how participants’ significant others influenced their behaviors, how participants evaluated the Internet environment, and how they judged their ability to search effectively.

## Materials and Methods

We conducted a qualitative study (Creswell and Creswell, 2017) to explore the experiences and perceptions toward using the Internet to search for health information among cancer survivors and their family members.

*Participants*

We recruited participants using a purposive sampling technique via the survey institute, Korea Data Network based on the following inclusion criteria: (1) Participants had to be 20 years of age or older, and (2) used the Internet to search for health information five times or more per week. The second inclusion criterion was particularly important to investigate experiences such as facilitation and disability factors in the use of health information. In total, thirty-one individuals agreed to participate; ten had been diagnosed with and received treatment for cancer, while the remainder had a parent who had been diagnosed with and received treatment for with cancer. Participants were aged 34 to 70 and grouped into three age categories. Table 1 presents the essential demographic characteristics and frequency of weekly internet searches for health information. 

*Materials and procedures*

Three focus group interviews (FGI) were conducted with participants in groups categorized according to age. For these interviews, we developed a semi-structured interview guide based on the TPB. This guide included open questions concerning participants’ mindsets (attitudes) toward searching health information on the Internet, opinions of significant others about searching health information on the Internet (social norms), and how participants deemed their ability to search for health information online (perceived control). At the end of the FGI we clarified and confirmed participant responses if the meaning of what they said was unclear. Interviews were conducted in equipped rooms for FGI and lasted about 90 minutes. Each interview was video-recorded and transcribed by a professional transcriber.

*Data analysis*

We followed a directed content analysis strategy (Hsieh and Shannon, 2005) to explore why people search the Internet for health information based on Azjen’s TPB (Azjen, 1991). Two authors (Jo & Kim) read all transcripts and selected descriptions for each concept (attitudes, social norms, and perceived control) of the TPB . Subsequently, themes for each concept were chosen and compared. To ensure the rigor of qualitative data analysis (Lincoln and Guba, 1985), the first author (Shin) reviewed and confirmed each process. Discrepancies were discussed until agreement was reached. Finally, themes and selected descriptions were classified into six themes based on the constructs of the TPB discussed in the following section.

## Results

The findings from the qualitative analysis indicated that study participants used the Internet in order to obtain health and medical information, verify credentials and institution details (i.e., ranks of doctors or hospitals), research health-related outcomes, and to communicate with cancer survivors. In addition to professional websites, participants searched for and consulted personal blogs or clubs, books, and articles.

The following paragraphs discuss each theme and quote based on the theory of planned health behavior (Ajzen, 1991).

*Attitudes*

Study participants were asked to comment on their perception of why they searched for health information on the Internet. Participants mentioned that they were motivated to search for information due to the lack of information provided about their course of care or to verify the information received from the doctor. They provided two reasons for their research: Fulfilling unmet knowledge needs and seeking confirmation through second opinion.

*Fulfilling unmet needs from doctors*

During their cancer treatments, participants had many questions but could not reach their doctors for answers. For example, some chemotherapy medications came with side-effects which made adherence to this treatment difficult. However, participants were unable to discuss with their doctors whether they continue with the prescriptions or stop.

“*To the same medication, people respond differently. We need to know*

*whether we need to stop or stick to it. But there is no one to reach to get an*

*answer from. I’m so frustrated*.”

Also, participants tried to introduce preventative management but criticized the lack of access to doctors when seeking health information for prevention.

“*I can see the doctor only when I’m sick. I can’t see the doctor just for asking*

*preventive things. But I need to take care of myself, my health, so I need to go*

*online*.”

*Participants discussed having bought functional foods from the Internet or home*

shopping websites, but they could not decide whether these foods provided the required health benefits to maintain their health. Further, new functional foods were regularly advertised; consequently, participants found it challenging to choose whether they should finish the one they had already bought or whether the new one would be better for them.

“*I bought root extract and took it for one or two months, then I bought red ginseng and took it one or two months, and then policosanol. I was a fool. They are just supplements. I need to have a healthy lifestyle, such as doing exercise, a low salt diet etc. But as you know, it is not easy to stick to this alone in daily life*.”

After repeating these cycles of consuming functional foods, participants finally admitted that they needed to modify their lifestyle in order to maintain their health. These modifications are not easy to do alone, so they asserted that they needed support from health professionals who could provide specific, feasible, and individually tailored strategies that they could apply in their daily lives. If they felt they could not get this support, they went online and searched for health information.

*Confirmation through second opinion*

Participants added that they “*go online*” because they wanted to countercheck doctors’ opinions. They believed that physician induced demand might motivate practitioner doctors to pay attention and make decisions in order to make a profit. Therefore, they believed there would be a high probability of overdiagnosis or unnecessary treatment. Participants discussed experiences from acquaintances being over-diagnosed in local clinics or general hospitals and therefore preferred to consult doctors in university hospitals. However, the long wait-time for appointments in university hospitals and the short duration of consultations led to frustration among participants. Consequently, participants felt forced to go online.

“*They have to think about money because they have to run their business. So, we should not believe what they say but look for another doctor in any university hospital. Doctors in university hospitals are hired by these hospitals, so they don’t need to worry about business things. But (in this case), they are too busy, and it is tough to get reservation. Even when we do meet, they don’t have time to explain things thoroughly*.”

*Social norms*

Ajzen (1991) proposed that when people received support from individuals important to them, they are more likely to follow through with intended behaviors. Therefore, we asked participants how their significant others viewed their use of the Internet to search for health information. Participants received positive feedback from their significant others; this included key phrases such as “*a required common step for sure among patients*” and that it was intended for “*helping each other.*”

*A required common step for sure among patients*

Participants reported that once they received a medical diagnosis, going online to get related.

health information was “*a common and even required step*” for them to share their experiences. All participants agreed that they needed second opinions as well as specific information they thought their doctors would not provide. They felt lucky if they had health professionals as friends or relatives because they could ask them, and this made their search easy. Limited access to health professionals, due to geographic or other financial reasons, were the most common reasons for participants to search for health information on the Internet.

*Helping each other*

Internet sites have become places for sharing experiences and information among similar people. In order to be a member of cyber communities such as the “cancer patient union or survivor union,” people need to register, and in some cases, pay for a membership. Participants reported that they were willing to pay for memberships and the support they obtain through these communities. They also discussed that they wished health professionals were among the members of these communities to facilitate fast and reliable answers.

*Perceived control*

Questions that aim to determine a person’s perceived behavioral control, typically entail a request to describe what makes the behavior difficult or easy to execute (Fishbein and Ajzen, 2010). Participants responded searching the Internet was challenging because it was “*difficult to choose because of overwhelmed with information” and “difficult to search because of difficulty in the search environment*”. 

*Overwhelmed with information*

Participants’ first impression of using the Internet for health information was that it is effortless to go online and access information. However, since they were not health experts, they had difficulty choosing appropriate information sources. When participants were asked to what extent they followed recommendations provided in the information obtained from the Internet, they complained about the amount of information and the difficulty to distinguish professional information from advertisements.

“*There are too many advertisements, too many. It is hard to tell which one is*

*good and which one is selling products*.”

In particular, when keywords of interest are entered into search engines, they were often linked to commercial advertising sites, which are often difficult to distinguish from professional sites with reliable information. Some advertisements started with professional information with references but, at the end of the article, promoted related health functional foods or supplements.

“*The advertisements become sneakier. They look like professional news but in fact it was an advertisement. They combined news and advertisements in one screen. Then it is hard to ignore. Advertisements are everywhere. Why don’t we have any regulation for that?*”

Participants reported that they hoped governments would provide reliable websites with practical guidelines, so they would not get frustrated by spending so much time searching and not finding the needed information.

*Searching Milieus*

The participant complained about the difficulties in the search environment. They mentioned that, in many cases, it is difficult to understand the cancer information because it contains complex medical terminology, or the text is small and difficult to read, and the content is too large. 

“*Well, if looking on results it is actually not easy to find customaries information. I have difficulties to find exactly the desired information because it is necessary to handle a lot of information*”

“ *In my case, I only use ‘Daum’. Other search sites didn’t fit me well because the writing was small. I can’t read the information from those sites because letters are too small and need use full screen*.”

**Table 1:** Characteristics of Study Participants

Characteristics	Participants (n=31)
Gender	
Male	17 (55%)
Female	14 (45%)
Age group	
20s and 30s	10 (32.3%)
40s and 50s	11 (35.5)
60s or older	10 (32.3%)
Employment status	
Employed	14 (45.2)
Self-employment	6 (19.4%)
Unemployment and Retired	11 (35.5%)
Frequency of health information searching	
1 to 4 times a week	4 (12.9%)
5 times a week or higher	27 (87.1%)
Frequency of cancer information searching	
1 to 2 times a week	8 (25.8%)
3 to 4 times a week	8 (25.8%)
5 times a week or higher	15 (48.4%)

**Figure 1:**
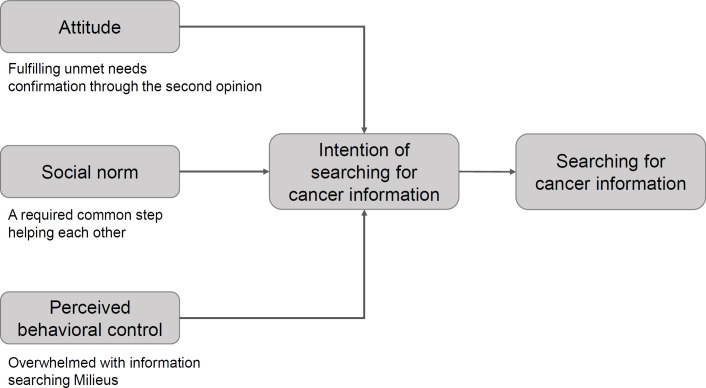
Framework of Six Themes based on the Theory of Planned Behavior

## Discussion

This study aimed to explore the cancer survivor and their family’s experiences of searching for health information related to cancer on the Internet. Using a qualitative approach, we obtained individual vivid experiences of patients and their family members that are difficult to achieve using quantitative methods. The main findings of the interviews with participants emphasized a strong incentive to seek health information to protect their health, indicated by comments such as “*I protect my health.*” The main reason for searching for health information was their belief that their health was their own responsibility. This initiative was motivated by the need to address the dissatisfaction of receiving partial or limited information from medical personnel, to share their experiences and help other patients, and to overcome difficulties in obtaining and evaluating medical information. Wagner (1998) emphasized the importance of an informed, activated patient in the Chronic Care Model. Previous studies have shown that health literacy provides a basis for improving self-management skills for chronic diseases, including cancer. For example, in a systematic review of 31 papers that assessed the relevance of health literacy for self-management of chronic disease, Mackey et al., (2016) found that high levels of health literacy were positively associated with high levels of health care knowledge and self-efficacy. 

The results of our study showed that cancer survivors and their families were not satisfied with the information received from their doctors. They thought the information needed to be confirmed using other evidence sources. These findings are similar to other studies of cancer patients and the general population. A recent scoping review of 117 articles published between 2007 and 2017 on cancer-related health information, revealed that patients had the highest need for information on prognosis and treatment. In contrast, non-patients had the highest need for general cancer information, prevention, and cancer examination. Caregivers sought information about treatment, psychosocial support, and prevention (Jo et al., 2019). Also, they wanted not only medical but also psychosocial support information. The results from previous studies and the current study highlight the importance of cancer information as a complimentary service to those already provided by medical personnel. Furthermore, it emphasizes the necessity to produce information resources that will satisfy patients’ needs. The authors made a fait accompli that information sharing among cancer survivors was a very natural process where they helped each other. We supposed that the reason this information sharing occurs relates to the information coming from laypeople; who have experienced a similar situation, have gained knowledge, and also harbors an expectation to give and receive emotional support. However, it should be noted that social media platforms, groups, and networks need to be managed to prevent it from being shared for commercial purposes. 

One of the most common reasons consumers use social media is to share health-related information with acquaintances, such as friends and relatives. However, this can lead to misleading information without scientific evidence being distributed and accepted without verification because it was received from a close network (El Sherif et al., 2018). Research about questioning reliable assessments of health information on social media recommends that health information retrieved through social media should be evaluated differently than other online health information (Dalmer, 2017). For example, internet-based technologies such as patients’ portals and apps managed by health care institutions are proven to be effective for cancer care coordination (Mattsson, 2017). Nevertheless, not all online health-related communities are managed by qualified professionals or government bodies in South Korea. Disseminating reliable information with easy access could be done via online communication systems, thus enabling health coordinators to ensure cancer survivors and their family members receive qualified information. 

Issues related to health functional foods were crucial. All the participants had experienced of buying two to three functional foods without consultation with health professionals. In the literature, more than one-third of Korean citizens, 20 years and older, had purchased health functional foods from commercial advertisements (Ohn and Kim, 2012). Since 2002, the Korean government regulates advertisements for health functional foods such as using certification marks and noting adverse effects (Ministry of Food and Drug Safety, 2018). The results of this study suggest that these regulations need to be reinforced on the Internet. 

The discussion about difficulties experienced with choosing relevant sites and the challenges with screen design, such as text size, highlights the perceived behavior control element where participants felt overwhelmed with information. The results of the interviews underscore the importance of knowledge transformation. Particularly, improving the online environment for individuals searching for cancer information with a focus to increase health literacy. There is a need to provide a navigation function to, more efficiently, search for necessary and evidence-based information. Recently, the effect of infographics has been emphasized as a way to effectively connect large amounts of data and complex information (Jo et al., 2017). 

Study participants possessed positive attitudes toward searching the Internet searching for health information because they were willing to take responsibility for their health, a key factor for person-centered care. They desired to be informed and actively involved in the decision-making process concerning cancer care and wanted access to health information related to disease prevention. Given these reasons, roles of primary health care need to be extended to cancer survivorship, and establishing reliable online communication systems between cancer survivors and the health profession would be beneficial.

## Fundings

This work was supported by the National R&D Program for Cancer Control, Ministry of Health and Welfare, Republic of Korea [No. 1720250].
